# Stem Cells: The Game Changers of Human Cardiac Disease Modelling and Regenerative Medicine

**DOI:** 10.3390/ijms20225760

**Published:** 2019-11-16

**Authors:** Elvira Immacolata Parrotta, Stefania Scalise, Luana Scaramuzzino, Giovanni Cuda

**Affiliations:** Department of Experimental and Clinical Medicine, Research Center for Advanced Biochemistry and Molecular Biology, University “Magna Graecia” of Catanzaro, 88100 Loc., Germaneto, Catanzaro, Italy; stefania.scalise@unicz.it (S.S.); scaramuzzino.luana@unicz.it (L.S.); cuda@unicz.it (G.C.)

**Keywords:** pluripotent stem cells (PSCs), embryonic stem cells (ESCs), induced pluripotent stem cells (iPSCs), disease modelling, regenerative medicine, cardiovascular disease (CVD), heart failure (HF)

## Abstract

A comprehensive understanding of the molecular basis and mechanisms underlying cardiac diseases is mandatory for the development of new and effective therapeutic strategies. The lack of appropriate *in vitro* cell models that faithfully mirror the human disease phenotypes has hampered the understanding of molecular insights responsible of heart injury and disease development. Over the past decade, important scientific advances have revolutionized the field of stem cell biology through the remarkable discovery of reprogramming somatic cells into induced pluripotent stem cells (iPSCs). These advances allowed to achieve the long-standing ambition of modelling human disease in a dish and, more interestingly, paved the way for unprecedented opportunities to translate bench discoveries into new therapies and to come closer to a real and effective stem cell-based medicine. The possibility to generate patient-specific iPSCs, together with the new advances in stem cell differentiation procedures and the availability of novel gene editing approaches and tissue engineering, has proven to be a powerful combination for the generation of phenotypically complex, pluripotent stem cell-based cellular disease models with potential use for early diagnosis, drug screening, and personalized therapy. This review will focus on recent progress and future outcome of iPSCs technology toward a customized medicine and new therapeutic options.

## 1. Introduction

**Figure 1:**
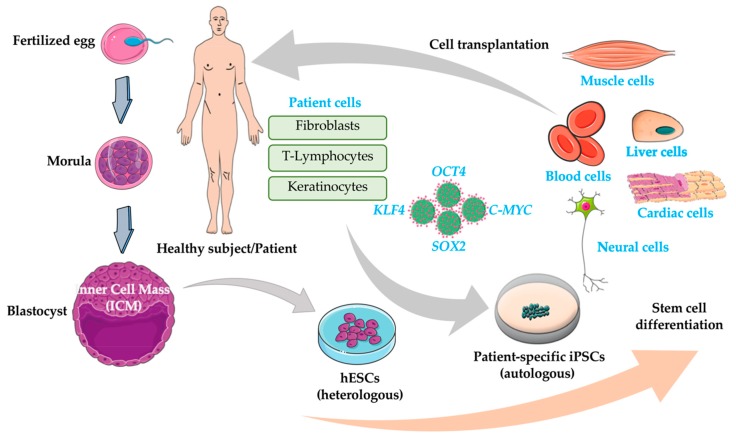
Pluripotent stem cells for cell transplantation therapy.

Cardiovascular diseases (CVDs) refer to a group of disorders affecting the heart and blood vessels and are the main cause of death worldwide. CVDs are considered multi-faceted disorders, since both genetic and environmental factors (obesity, diabetes, smoke) play a fundamental role in their onset and progression. Patho-physiologically, CVDs are associated with heart dysfunction and cardiomyocytes (CMs) death, generation of fibrosis and scar tissue, and abnormal ventricular remodeling [1]. Cardiomyocytes loss is an irreversible process which is often followed by scar formation; these phenomena, taken together, represent the two main pathological problems since cardiomyocytes loss is not replaced by new ones because the cardiac tissue has a very limited regenerative capacity, while scar/fibrosis leads to heart failure (HF) or dilated cardiomyopathy (DCM). Currently available therapies improve symptoms and slow down the pathological cardiac remodeling but fail to address the cardiac tissue repair [2]. A comprehensive understanding of the molecular mechanisms underlying cardiac diseases is the key for the development of new and effective therapeutic strategies. On the other hand, the lack of biological relevant and robust disease models has limited the progress in therapeutics. Most of our knowledge about the molecular basis of CVDs has come from rodents that, although very useful, do not accurately mirror human diseases because of their fundamental biological, genetic, and electrophysiological differences. Primary human cardiac cells (both healthy and disease-bearing) are challenging to isolate and propagate in culture for the time required for examination. Pluripotent stem cells (PSCs) are undifferentiated cells having the ability to undergo unlimited self-renewal and to give rise to derivatives of all three developmental germ layers (ectoderm, mesoderm, and endoderm) [3] and even to germ cells with the potential to regenerate an entire organism [4], both *in vivo* and *in vitro* (Figure 1).

**Figure 2:**
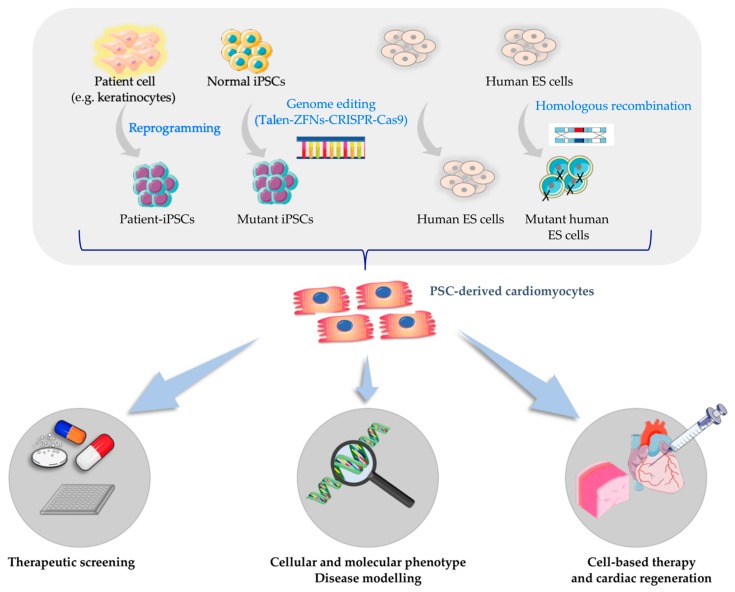
Biomedical applications of human pluripotent stem cells.

These properties make PSCs a precious and irreplaceable platform for a variety of biomedical applications, such as the study of early stages of development biology [5], disease models [6,7], drug screening and toxicity testing [8], cell transplantation and regenerative medicine [9] (Figure 2).

Based on this knowledge and potential, human embryonic stem cells (hESCs) became rapidly and progressively more and more exiting since they were first isolated in 1998 [10]. Although very fascinating, the use of human ESCs is hampered by various limitations: (i) their derivation implies the destruction of the embryo arising significant ethical controversies [11]; (ii) despite the possibility to create mutated ESC lines to induce specific disease causal mutations, they often fail to fully recapitulate the disease phenotype seen in patients [12], and finally, (iii) their potential use as cell therapy is hampered by the risk of immune response and consequently rejection due to their allogenic nature. The discovery, in 2007, that adult cells can be successfully reprogrammed into pluripotent stem cells (named induced pluripotent stem cells, iPSCs) has represented a milestone in stem cell biology and has emerged as an extraordinary new platform to overcome all the limitations associated to the use of animal models and hESCs. iPSCs were firstly generated by virus mediated overexpression of four transcriptional factors (OCT4, KLF4, SOX2, and c-MYC) into human fibroblasts [13]. Other studies have later reported the generation of iPSCs from other somatic cells and using a different reprogramming cocktail [14]. Compared to mutated ESCs, iPSCs have two major advantages: (i) no ethical issues arise from the generation and use of iPSCs since they are derived from somatic cells, and, (ii) they maintain genomic and epigenomic profiles of the patient’s they are derived from. Here we discuss the role of human pluripotent stem cells (PSCs) as new players in modeling cardiac disease in vitro and in future perspective of personalized and regenerative medicine.

## 2. Cardiac Disease Modeling

Disease modeling has relied mostly on the use of mouse models genetically modified for knockout or disease-specific mutations in the gene of interest. Despite animal models have provided interesting information, mice are genetically different from humans and may not provide a comprehensive knowledge on how mutations affect the onset and the development of human disease. Understanding the molecular basis of disease has allowed the identification of targets and signaling pathways that might represent potential candidates against which scientists can develop new therapeutic strategies. Moreover, therapeutics that showed encouraging results in animals often did not provide any improvement in humans. The lack of human cellular models for disease modeling has delayed our know-how regarding the molecular mechanisms underlying disease and even more the possibility to discover effective cures for currently untreatable disorders. Human stem cells-based disease models offer the advantage for a more refined comprehension of disease mechanisms that in turn is the way to unveil new therapeutic targets. Modeling early onset childhood disease results very successful because stem cells allow to faithfully recapitulate phenotype during early stage of differentiation [15]. To date, iPSC models have been used to model a large number of genetic cardiac diseases such as catecholaminergic polymorphic ventricular tachycardia, CVPT [16], arrhythmogenic right ventricular cardiomyopathy, ARVC [17,18] and many others. An early study of iPSC-based disease model of Long QT syndrome Type 1 (LQT-1) successfully recapitulated the clinical features of the disease in iPSC-derived cardiomyocytes from patients [19]. Using iPSC technology-based model disease, another study reported that the change of heart beat rate at early disease onset represents a cure for patients with long QT syndrome (LQTS) [20]. There are also reported cases of neurodevelopmental disorders in which the cardiac function is involved and negatively affected. An example of such disorders is the Williams-Beuren syndrome (WBS), a rare genetic neurodevelopmental disorder that causes cardiovascular disease. An iPSC-based model of WBS was generated and iPSCs were coaxed to differentiate into smooth muscle cells (SMCs) since the patient was affected by aortic and pulmonary stenosis. Intriguingly, iPSCs-derived SMCs revealed an immature proliferative phenotype accompanied by a reduction of contractile and functional properties compared to SMCs from a healthy individual [21]. Using iPSC-based cellular model for single gene disorders is quite straightforward but a great variety of cardiovascular disorders often involve multiple genes variants and, in some cases, also different tissues and cell types; moreover, many cardiac diseases are characterized by a late onset. Using iPSC-based models for such complex diseases remains still challenging. Carvajal-Vergara and collaborators succeeded in the generation of an iPSC model from patients with Leopard syndrome for which a major phenotype is hypertrophic cardiomyopathy that was shown in iPSC-derived cardiomyocytes and provided molecular insights into signaling pathways that might be involved in the disease phenotype promotion [22].

## 3. Cardiac Cell Therapy: Variety of Stem Cell Types Investigated

Despite significant advances in prevention, diagnosis, and treatment of CVD, in the most severe cases there is no other way than heart transplantation. Numerous strategies have been developed and various cell types have been investigated to serve as potential source to replace damaged or dead cardiomyocytes. Stem cell therapy (SCT) represents a promising way addressed to the treatment of ischemic cardiomyopathy and is the only alternative to heart transplantation [23]. Cell therapy entails either transplantation of exogenous therapeutic cells directly injected into the patient’s heart or by activation the endogenous regenerative processes through stimulation of adult tissue-restricted stem cells. Adult stem cells are found in different organs and tissues in which they play a role in maintaining tissues homeostasis. A wide spectrum of stem cells at different developmental stages has been investigated to test their potentiality to improve cardiac function through heart tissue regeneration which represent the final long standing ambition [24]. The first stem cell-based preclinical results showing the repair of infarcted cardiac tissue were reported in 2001 [25]. Despite the extraordinary efforts in this field, up to now there are no effective cell-based therapy for cardiac diseases. The therapies currently available are palliative and useful only to reduce the scar formation and the pathological heart remodeling [26] but poorly or not at all effective on cardiomyocytes loss for which heart transplantation is needed. Among the variety of cell types investigated for cardiac therapy, skeletal myoblasts (SM), derived from satellite cells and localized under the basal lamina of skeletal muscle [27], were the first ones tested. SMs are highly proliferative, resistant to ischemia, capable to give rise to myotubes and to form functional muscle-like cells grafts in damaged myocardium [28]. Despite these interesting features, SMs are unable to acquire a cardiomyocyte-like phenotype and to form electrical junctions and often give rise to ventricular arrythmias [29,30,31,32]. Intramyocardial injection of SMs in patient with ischemic heart disease did not produce any significant improvement of left ventricle function [33], rather induced arrythmia after cell transplantation [34]. Bone-marrow (BM)-derived cells were successfully used for intramyocardial injection in a murine model of myocardial infarction (MI) [35]. The injection of autologous BM-derived mononuclear cell (MNCs) in MI patients significantly decreased the infarct region with neovascularization and myocardial regeneration [36]. BM-derived cells can trans-differentiate into functional cardiomyocytes [37] beyond their known ability to become endothelial and smooth muscle cells. Other studies have instead disproved the hypothesis of a cardiogenic potential of these cells [38], suggesting that the apparent beneficial effect may be due to a fusion with resident cardiomyocytes and not to an effective role of BM-derived cells [39]. Epithelial stem cells (ESCs) are a pro-vascular subpopulation of hematopoietic stem cells (HSCs) identified by the expression of CD24 and CD133 surface markers [25]. CD133^+^ cells have been used in clinical trials for autologous transplantation in patients with MI showing an improvement of cardiac perfusion and left ventricle function during the short-term follow-up; unfortunately, long-term follow-up did not reveal any cardiac advantage [40]. Based on their immunosuppressive properties, mesenchymal stem cells (MSCs) have aroused great interest for their potential use for allogenic transplantation [41]. Unfortunately, the use of MSCs in cardiac regeneration still remains controversial and inconsistent: some studies have shown a beneficial effect on cardiac function after intracoronary injection in patients with acute MI [42], while others have greatly reduced the impact of MSCs to improve heart function [43]. MI is usually followed by a massive mobilization of circulating stem cells and progenitors suggesting a potential role of these cells in heart tissue healing [44]. Strategies to improve and optimize protocols for a higher retention of cells injected into MI hearts are currently under investigations and, indeed, very recently it has been documented a promising approach in scar reduction and an improvement in survival of transplanted MSCs when these cells are co-delivered with stem cell derived exosomes [45]. Adipose-derived stem cells have been reported as successful in heart failure therapy [46] but again, also in the case of adipose-derived stem cells, there are conflicting results [47]. Overall, all the described stem cell types resulted effective in a short time but fail to restore the cardiac physiology in a permanent manner. The incapacity to efficiently maintain a long-term beneficial effect might be due to the fact that these cells are poorly represented and have limited differentiation potential.

## 4. Cardiac Adult Cells and Progenitors

For a long time the adult heart has been considered an organ with any regenerative capacity. This wrong belief has changed with the identification of cardiac progenitor cells that have the function to maintain a certain degree of cardiomyocytes turnover [48]. During the past decade, different cardiac stem cells (CSCs) and progenitors such as cardiosphere-derived cells (CDCs) [49], stem cell antigen (Sca-1^+^) [50], insulin gene enhancer protein (Isl1^+^) [51], c-Kit^+^ cells [52], have been identified. Cardiac adult cells have been used in different studies of animal models showing an improved myocardial performance [53,54]. Wang and collaborators have shown that multiple injections of CDCs derived from infants can significantly improve cardiac functions in dilated cardiomyopathy (DCM) rat models compared to adult CDCs [55]. CSCs injection into infarcted hearts have shown a certain capacity to heal damaged cardiac tissue by both *de novo* differentiation and cell fusion with resident host cardiomyocytes [56]. Altogether, these and other studies support the potentiality of adult stem and progenitor cells to acquire a cardiovascular phenotype within the cardiac environment and that this acquisition might be, at least partially, associated with the release of biologically active cytokines and growth factors such as transforming growth factor β (TGF-β), vascular endothelial growth factor (VEGF), and epidermal growth factor (EGF), responsible for neovascularization, recruitment and activation of resident progenitor cells, and reduction of cardiomyocytes death [57,58]. Unfortunately, controversial results and data seen for the majority of stem cells described here involve also cardiac stem and progenitor cells [59]. The incomplete success of adult stem cells and progenitors in stem cell-based cardiac therapy is also determined by the important technical challenges in their isolation from tissues of origin and lack of optimized culture conditions.

## 5. Pluripotent Stem Cells

The ability of adult stem cells to repair damaged myocardial tissue has been mainly evaluated in preclinical models but only a few of them have reported promising data and were further employed in clinical trials. Apart from their safety, adult stem cells only showed marginal beneficial in cardiac regeneration capability pointing to the need to develop new therapeutic strategies.

A potential novel method is the possibility to generate cardiomyocytes from pluripotent stem cells (including embryonic stem cells, ESCs and induced pluripotent stem cells, iPSCs) that, compared to other cell types employed in clinical trials and animal models, are master cells with master properties such as self-renewal and capability to evolve into cells and tissues of the three primary germ layers. Self-renewal is a property owned of specific cells that can divide indefinitely and form cell progeny indistinguishable from it. Under the right circumstances, a PSC can produce specialized cell types, both in vivo and in vitro. After this development stage is over, the stem cell-derived differentiated cell no longer has the potential to develop further. Besides their potential to give rise to all different cell types, PSCs have the advantage to be expanded and cultured for long time without losing their pluripotency. ESCs are pluripotent stem cells derived from the inner cell mass early stage embryo. The first human ESC line was isolated in 1998 by Thomson and collaborators [10] and has immediately provided the chance to obtain a renewable source of healthy cells to treat a wide range of disease such as heart diseases, based on their potential to develop into specialized cells that can ultimately replace diseased cells and tissues. Studies based on animal models revealed that cardiac environment can trigger itself the differentiation of ESCs into functional cardiomyocytes that can in turn replace damaged or lost cardiac cells. This process occurs for the release of paracrine factors such as transforming growth factor β (TGF-β) superfamily [60]. Moreover, ES-derived cardiomyocytes electromagnetically integrate into the host myocardium where they positively influence the cardiac function and remodeling. PSC-driven cardiac regeneration involves various molecular and cellular processes that end with the recovery of cardiac homeostasis and function [61]. Chong et al provided the first clinical transplantation of human PSC-derived CMs injected into the heart of adult pigtail macaques after MI. These authors showed that a great number of cells grafted in non-human primate (NHP) and electrically coupled with the host heart over a period of three months. Additionally, investigators also reported an improvement of cardiac function [62]. The effectiveness of human PSC-derived cardiovascular progenitor cells (CVPCs) has been tested in an NHP model. Authors first induced MI in cynomolgus monkey by permanent ligation of the left anterior descendent artery and 30 minutes later they injected 10 million of iPSC-CVPCs into myocardium. This study documented a recovery of cardiac function but any transplanted cell was detected after treatment with immunosuppressive drugs [63]. An elegant model of beneficial and positive outcome derived from the use of ESCs in cardiac therapy has been described by Menaschè and collaborators. In this study, ESCs were first treated with BMP2 and SU-5402 (an inhibitor of FGF receptor) and subsequently electromagnetically sorted for stage-specific embryonic antigen (SSEA)-1 expressing the cardiac transcription factor Isl-1. Enriched Isl1^+^ cardiac progenitor cells were embedded into a fibrin scaffold and injected in patient with severe HF. The procedure reported a 10% increase of the left ventricle ejection fraction (LVEF) and improvement of the symptoms without complications such as or tumor formation [64]. Despite promising and encouraging regenerative potential, the use of human ESCs leads to important ethical concerns making their application highly debated and controversial [65].

**Figure 3:**
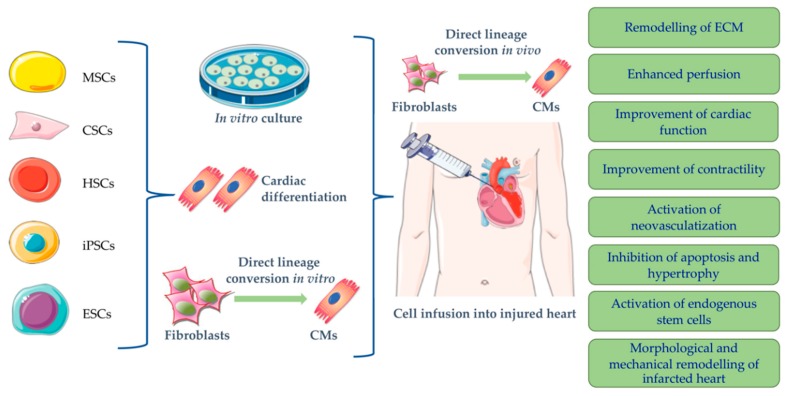
Cardiac regeneration.

The discovery, in 2007, that pluripotent stem cells can be generated from adult somatic cells has revolutionized stem cell biology and opened a great interest in their potential therapeutic application in heart diseases [66] offering an unprecedented opportunity to study human disease [7] at the cellular level and for a customized PSC-based therapy. Unlike ESCs, iPSCs are patient-specific, and thus overcome the problems related to allogenic nature and risk of immune rejection. Additionally, being patient-specific, iPSCs represent a robust platform for in vitro disease phenotype profiling providing new insights into the disease etiology with the possibility to identify new therapeutic strategies [67,68]. Patient-specific iPSCs provide unlimited disease-relevant cells and serve as cell source for cardiac regenerative applications after the rescuing the casual mutation taking advantage from homologous recombination technologies. Allogenic iPSC-based cell therapy has emerged as promising strategy since the iPSCs banks offer the possibility to select human leucocyte antigene (HLA)-matched iPSCs lines. Moreover, allogenic transplantation of nonhuman iPSC-CMs have shown to greatly enhance cardiac contractile system in a nonhuman primate model of MI [69]. An overview of stem cells types investigated for a potential use in cardiac therapy is shown in Figure 3.

## 6. Direct Reprogramming of Somatic Cells into Functional Cardiomyocytes

The concept of cellular reprogramming has been further improved with the manipulation of cellular identity as shown by the direct conversion of fibroblasts to cardiomyocytes without going through an intermediate state in a process named direct differentiation or trans-differentiation. Srivastava and collaborators have demonstrated the generation of functional cardiomyocytes by retroviral delivery of three cardiac transcription factors, Gata4, Mef2c, and Tbc5 (GMT) into murine fibroblasts. The same author used genetic lineage tracing to demonstrate that non-myocytic cells in murine heart can be converted into cardiomyocytes by in vivo delivery of GMT after coronary ligation and that this strategy has the potential to reduce the infarct size [70]. This process has a high impact since bypassing the pluripotent state might generate more efficient and mature CMs and decreases the tumorigenic risk associated with the use of pluripotent stem cells. The potential tumorigenicity risk is one of the major stumbling steps in iPSC-based cell replacement and it is associated with undifferentiated iPSCs in the cell population that will be used in regenerative transplantation. The use of viral vectors for direct cell conversion limits the application of this strategy in clinical practice.

## 7. From 2D to 3D

Cell engraftment after myocardial injection still remains a big goal to be achieved. The presence of less expandable scar areas compromises the viability of injected cells and advances in tissue engineering are currently oriented to the generation of heart tissues that can be implanted onto the infarcted region. Different extracellular matrix (ECM) such as Matrigel, fibrin, and collagen are currently under investigation. Tissue engineering aims to the generation of 3D cardiac tissue with a complex ECM structure similar to that of native human heart [71]. Three-dimensional human heart constructs consisting of hiPSCs-derived CMs and endothelial cells were used to repair large cardiac defects in guinea pig hearts demonstrating a reduction of pathological remodeling, enhanced electrophysiological properties, myocytes proliferation, vascularization, and electrical coupling in the intact heart [72]. Recently, a fibrin-based scaffold using human iPSC-derived CMs and -smooth muscle cells was developed [73]. The optimal combination of scaffold material and cell types is still in its developmental way and strategies that can guarantee the survival and health of transplanted cells, neo-angiogenesis and reduction of apoptosis need to be further developed.

## 8. Limitations of iPSCs Technology

Despite the morphological and functional similarities between iPSCs and ESCs, different studies have also reported the existence of important dissimilarities [74,75,76]. It becomes thus necessary to continue the investigation of the mechanisms underlying pluripotency and identify crucial transcription factors, molecules, and signaling pathways that might have a role in enhancing human pluripotency [77]. Differently from ESCs, iPSCs do not arise ethical controversial neither adverse immunological reaction since they are patient-specific and their future application for regenerative medicine is intended, at least for some diseases, to be used for the patient they are derived from. Despite their promises, the optimal integration of iPSC-derived cardiomyocytes in the host tissue and their capacity to improve cardiac function in animal models of MI [78,79], before iPSCs can be used for human cell therapy a series of problems, such as genomic instability, interline variability, risk of teratoma formation, and low maturation and of their derived cardiomyocytes need to be solved. The karyotype assessment of iPSCs and their derivatives is a crucial requirement that needs to be addressed prior to their use in cell therapy [80]. iPSCs suffer of genomic instability and epigenetic memory associated to the reprogramming process that make wonder if this cell system and their differentiation derivatives are epigenetically influenced by external factors [81]. Yoshihara and collaborators, for instance, have described unusual methylation patterns and mutations either associated to variants in parental somatic cells or that take place through the reprogramming process or are due to culturing time in vitro [82]. Teratoma formation in recipients is a primary concern and major side effect of iPSCs transplantation [80]. The incomplete maturation of their derivatives [83,84] is reported by vast literature showing that iPSC-derived CMs are structurally, functionally, and genetically similar to embryonic CMs pointing to the need to discover strategies to make the maturation of iPSC-derived CMs more closer to the level seen in adult human heart [85]. The generation of immature iPSCs derivatives could be partially explained by the lack of a 3D environmental cues [86]. The incomplete maturation has important implications for the use of iPSC-CMs in a model disease setting since this might mask the real disease phenotype with a potential loss of information; consequently, not only the disease phenotype is not accurately mirrored, but it is also difficult to accurately test the efficacy/toxicity of new compounds. Despite the advances, the differentiation of iPSCs to completely matured cardiomyocytes in vitro still remains an unsuccessful process [87,88] and various signals are currently under investigation to allow complete and mature differentiation. A summary of prevalent cardiac differentiation methods in shown in Table 1.

Several studies have attempted to develop strategies able to generate more mature iPSC-derived cardiomyocytes [85]. Beyond low maturation potential, heterogeneity of iPSC-derived cardiomyocytes population currently represents another important step that needs to be further solved. iPSC-derived cardiomyocytes are in fact a mixture of three types of cells (ventricular-like, atrial-like, and nodal-like). The current differentiation protocols are inefficient since the final cell population is highly heterogeneous including non-CMs cells and a highly pure and mature CMs population is needed in cell transplantation therapy to ensure sufficient engraftment in the host myocardium where they also need to exhibit electrical and mechanical coupling with resident cardiac cells. For some studies, mixed population of cardiac and non-cardiac cells might still be acceptable, while others, such as those that are finalized to a cell-based therapy, need highly pure preparations of cardiomyocytes. Different transcription factors and critical signaling pathways are responsible for different cardiac subtypes differentiation [104] and recent advances are made to more precisely regulate cardiomyocyte lineage specification. It has been reported the beneficial effect of retinoic acid (RA) in embryoid bodies (EBs) to reduce the specification of ventricular-like cells CMs and increase the atrial population [105]. Overall, important further improvements of iPSC-derived cardiomyocytes in terms of maturity, cell specificity, and standardization of the culturing methods need to be reached to lead to a more comprehensive understanding of the molecular basis in cardiovascular diseases and for the future development of personalized and tailored medicine approaches. Very recently it has been established an unbiased proteomics method for hPSCs-CMs maturity evaluation and identification of new markers for maturation assessment [106] determining the optimal number of iPSC-CMs to be injected in the damaged myocardium. Finally, an adverse effect of cell therapy for CVDs is the arrhythmogenic phenotype graft-derived myocardium due to the lack of synchronous operation with resident CMs.

## 9. Genome-Editing Technologies

New advances are based on the combination of iPSC technology with genome-editing technology, such as zinc-finger nuclease (ZFN) or clustered regularly interspaced short palindromic repeats- (CRISPR-) associated 9 (Cas9) system. The genome editing technology enables the generation of genetically modified patient-specific iPSCs to elucidate gene function and mechanisms of human cardiac diseases or targeted gene(s) causing disease correction [107]. CRISPR/Cas9 facilitates the generation of isogenic iPSC lines either by correcting the mutations or insertion that differ from the parental-edited cells only at the genome-loci [108]. In this context, CRISPR/Cas9 technology provides the basis to elucidate how the cells misbehave when the causal mutation is reversed. The CRISPR/Cas9 technology was successfully used in an iPSC-based CMs model of arrhythmogenic right ventricular cardiomyopathy (ARVC) identifying *SCN5A* as causative of cardiomyopathy, while corrected cells showed normal channel activity [109]. Restoration of genetic mutations in patient-iPSC-derived CMs has the potential to use the corrected cells for implantation back into the patient. These techniques require extensive investigation before they can be used in clinical practice and, additionally, they might be coupled with improved strategies to eliminate any residual undifferentiated iPSCs in the final population of cells that need to be transplanted into the patient.

## 10. Conclusions

Despite substantial advances and exciting discoveries, cardiac regeneration still remains an unmet ambition. Investigating the mechanisms by which different stem cell types govern the regeneration of infarcted heart is of utter importance for the development and discovery of new therapeutic strategies. The advances in stem cell technology, together with the knowledge of the endogenous mechanism involved in organ repair, has revolutionized the basis of the progress of regenerative attitude. The possibility to use stem cells to repair damaged cardiac tissue involves indirect and paracrine mechanisms. The latter includes the direct cardiac differentiation of injected SCs and their integration into the myocardium to compensate loss of CMs or endothelial cells. iPSCs technology has become a powerful tool for investigating the developmental biology and the pathophysiological mechanisms of various genetic cardiac diseases. Understanding how diseases develop and which are the signaling pathways and mechanisms responsible opens the way for the discovery of new targets against which scientists can develop now curative molecules and new therapeutic strategies. Additionally, iPSCs have significantly evolved for developing strategies for their potential use in future cell therapy and regenerative medicine. Heart transplantation represents the end stage of severe cardiac failure: the procedure is not at all simple and many patients have no chance of getting cardiac transplantation because of very restricted accessibility of hearts donors and other complications involved. Stem cell-based therapy for cardiac regeneration thus represents a new potential way to overcome injured heart, but many significant concerns need to be further investigated and determined. The challenge is now the understanding how this technology can be translated to real clinical application through optimization of differentiation and purification steps. Often, the discoveries with the greatest therapeutic benefits present the most difficult challenges; this is particularly true for pluripotent stem cells. Researchers are attempting to learn ways of controlling the development process of pluripotent stem cells into the many different cell types in the human body. Another current challenge is that the cells used in research are rejected from a person’s body due to their immune system. iPSCs have the potential to be used for the treatment of many diseases but new strategies attempting to overcame their current technical issues need to be developed. When this is achieved than iPSCs can really represent the hope for many people suffering of cardiac diseases.

## Figures and Tables

**Table 1:** Summary of cardiac differentiation methods.

Method	Molecules for mesoderm and cardiac specification	Ref.
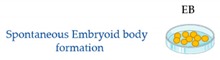	⮚ FGF2, FGF2, BMP4, VEGFA, Dorsomorphin, SB431542, DKK1	Kehat et al. 2001 [89]
	⮚ BMP4, Activin A, bFGF, VEGF, DKK1	Kattman et al. 2011 [90]
	⮚ FGF2, FGF2, BMP4, IWR1, Triiodothyronine	Yang et al. 2008 [91]
		Willems et al. 2011 [92]
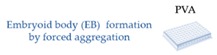	⮚ Activin A, bFGF, BMP4, SCF, VEGF, LI-BEL	Elliott et al. 2011 [93]
	⮚ BMP4, Activin A, bFGF, VEGF, DKK1	Burridge et al. 2007 [94]
	⮚ FGF2, FGF2, BMP4, IWR1, Triiodothyronine	Burridge et al. 2011 [95]
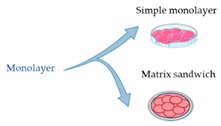	⮚ Activin A, BMP4, IWR1 or IWR4	Hudson et al. 2011 [96]
	⮚ Activin A, BMP4, FGF2, VEGFA, DKK1	Uosaki et al. 2011 [97]
	
	⮚ Activin A, BMP4	Laflamme et al., 2007 [98]
	⮚ Activin A, BMP4, FGF2, RAi, Noggin, DKK1	Zhang et al. 2011 [99]
	⮚ CHIR99021, Activin A, BMP4, XAV-939	Palpant et al. 2016 [100]
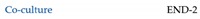	⮚ Insulin depletion, PGI_2_, p38 MAPK inhibition	Passier et al. 2005 [101]
		Graichen et al. 2008 [102]
		Freund et al. 2010 [103]
